# Coordinating a Team Response to Behavioral Emergencies in the Emergency Department: A Simulation-Enhanced Interprofessional Curriculum

**DOI:** 10.5811/westjem.2015.8.26220

**Published:** 2015-10-22

**Authors:** Ambrose H. Wong, Lisa Wing, Brenda Weiss, Maureen Gang

**Affiliations:** * Yale University School of Medicine, Department of Emergency Medicine, New Haven, Connecticut; † Bellevue Hospital Center, Adult Emergency Services, New York, New York; ‡ Bellevue Hospital Center, Hospital Police, New York, New York; § New York University School of Medicine, Department of Emergency Medicine, New York, New York

## Abstract

**Introduction:**

While treating potentially violent patients in the emergency department (ED), both patients and staff may be subject to unintentional injury. Emergency healthcare providers are at the greatest risk of experiencing physical and verbal assault from patients. Preliminary studies have shown that a team-based approach with targeted staff training has significant positive outcomes in mitigating violence in healthcare settings. Staff attitudes toward patient aggression have also been linked to workplace safety, but current literature suggests that providers experience fear and anxiety while caring for potentially violent patients. The objectives of the study were (1) to develop an interprofessional curriculum focusing on improving teamwork and staff attitudes toward patient violence using simulation-enhanced education for ED staff, and (2) to assess attitudes towards patient aggression both at pre- and post-curriculum implementation stages using a survey-based study design.

**Methods:**

Formal roles and responsibilities for each member of the care team, including positioning during restraint placement, were predefined in conjunction with ED leadership. Emergency medicine residents, nurses and hospital police officers were assigned to interprofessional teams. The curriculum started with an introductory lecture discussing de-escalation techniques and restraint placement as well as core tenets of interprofessional collaboration. Next, we conducted two simulation scenarios using standardized participants (SPs) and structured debriefing. The study consisted of a survey-based design comparing pre- and post-intervention responses via a paired Student *t*-test to assess changes in staff attitudes. We used the validated Management of Aggression and Violence Attitude Scale (MAVAS) consisting of 30 Likert-scale questions grouped into four themed constructs.

**Results:**

One hundred sixty-two ED staff members completed the course with >95% staff participation, generating a total of 106 paired surveys. Constructs for internal/biomedical factors, external/staff factors and situational/interactional perspectives on patient aggression significantly improved (p<0.0001, p<0.002, p<0.0001 respectively). Staff attitudes toward management of patient aggression did not significantly change (p=0.542). Multiple quality improvement initiatives were successfully implemented, including the creation of an interprofessional crisis management alert and response protocol. Staff members described appreciation for our simulation-based curriculum and welcomed the interaction with SPs during their training.

**Conclusion:**

A structured simulation-enhanced interprofessional intervention was successful in improving multiple facets of ED staff attitudes toward behavioral emergency care.

## INTRODUCTION

Patients with behavioral emergencies often present to the emergency department (ED) in acute agitation.^1^ The etiology of their agitation carries a wide differential diagnosis, including metabolic derangements, intracranial pathology, toxic and illicit drug ingestions and psychiatric emergencies.^2^ Care of these patients comes with innate safety risks for both the staff members and the patients themselves. EDs have been identified as high-risk settings for workplace violence (WPV).^3^ A national survey of emergency physicians found that at least one WPV act was reported in 78% of all responders, and 21% reported more than one episode.^4^ Several studies have shown that emergency nurses are at the greatest risk of experiencing verbal and physical assault as compared to nurses in other healthcare settings and to physicians.^5^,^6^ Precipitators of violence and aggression in the ED are most commonly attributed to alcohol/substance abuse, mental illness, and altered perceptions and confusion, all of which frequently exist in agitated patients.^7^,^8^ Management of acutely agitated patients consists not only of physical restraints and administration of appropriate medications but also utilization of de-escalation and agitation reduction techniques.^2^,^9^

Recent surveys of healthcare workers have identified a need for early communication of clear roles and responsibilities of hospital security and ED staff to improve safety during WPV events.^10^ Implementation of a structured team approach that promotes interprofessional collaboration to manage patients with behavioral emergencies has shown significant impact on mitigating aggression.^11^,^12^ In addition, improving providers’ attitudes and comprehension of factors contributing to patient violence has been directly linked to an improved workplace safety climate.^13^ However, staff members have expressed ongoing fear and anxiety when caring for potentially aggressive patients, even to the point where some providers intentionally avoided engaging patients and visitors whom they deemed to have violent tendencies in order to alleviate their stress symptomatology.^14^,^15^ Currently, educational strategies targeting WPV, including the widely adopted Nonviolent Crisis Intervention program from the Crisis Prevention Institute (CPI), have focused on an individual provider’s interaction with violent patients.^16^,^17^ Healthcare simulation provides a realistic but safe venue to address issues surrounding patient violence. More importantly, simulation–based education can both directly influence participant attitudes and encourage interprofessional teamwork due to its inherent ability to impact learners’ cognitive frames and promote peer-to-peer dialog during structured debriefing.^18^–^20^

Our study used a multi-modality, team-based approach to create a novel simulation-enhanced patient safety curriculum targeting staff attitudes toward patient aggression and interprofessional collaboration during the management of patients with behavioral emergencies in the ED. We assessed the potential success of the program through direct analysis of staff attitudes towards management of aggression with a validated survey instrument. Our hope was that this intervention would allow for a coordinated team approach that would improve safety for both patients and staff members.

### Assessment Instrument

We examined changes to staff attitudes as a result of this intervention via the Management of Aggression and Violence Attitude Scale (MAVAS), a published survey from a British nursing education group that has shown reliability and internal validity for assessment of staff attitudes toward patient aggression.^21^ Although this survey’s validation process was performed with a psychiatric patient cohort, transferability to the ED environment is feasible given that ED patients presenting with behavioral emergencies often carry psychiatric etiologies. Moreover, the survey authors included the psychiatric ED as one of the clinical environment for their investigation and thus allowed for applicability to the ED setting.^22^ The survey was subdivided into four constructs contributing to patient aggression: internal and biomedical factors of the patient; external and staff factors; situational/interactional perspectives; and staff perspectives toward management of patient aggression. This was distributed immediately pre- and post-session to assess the course’s direct impact on our staff. See Appendix 1 for a copy of the survey questions and elements. We used the paired sample Student’s *t*-test for our survey data analysis using IBM SPSS 21.0 software, and our study was approved by NYU School of Medicine’s Institutional Review Board as expedited review under the title, “Simulation-based Team Training for Care of Acutely Agitated Patients in the Emergency Department (i14-00846)” in May 2014.

## EDUCATIONAL OBJECTIVES

1. Describe and demonstrate effective interprofessional teamwork and communication skills to treat the patient with a behavioral emergency in the ED.
2. Identify roles and responsibilities of members of an interprofessional team that care for acutely agitated patients.
3. Display effective violence mitigation and de-escalation techniques.
4. Appropriately apply physical restraints and medical interventions during treatment of the agitated patient in the ED.
5. Demonstrate improvement in attitudes toward patients with behavioral emergencies through a better understanding of factors contributing to patient aggression.

## CURRICULAR DESIGN

As the care of the agitated patient requires balancing a complex range of clinical, communication and teamwork skills, we felt that applying David Kolb’s experiential learning theory as our educational framework would best suit our needs.^23^ We developed a simulation-enhanced interprofessional curriculum as the application of experiential learning where our physicians, nurses, patient care technicians and hospital police officers trained together to replicate the ED clinical environment. Content experts from the hospital’s Crisis Management Team (CMT) with extensive training in teaching de-escalation techniques and evidence-based management of aggressive persons joined us in our educational endeavor. They worked with physician, nursing and hospital police educational leadership to ensure that our curricular content was in line with current best practices from the literature.^2^,^3^,^17^ We used standardized participants (SPs) to maximize fidelity during case-based simulations that were designed to incorporate de-escalation and personal defense techniques, team-based interprofessional approaches, application of physical restraints and adjunctive medication route and dosing options.

### Didactics

**Figure 1:**
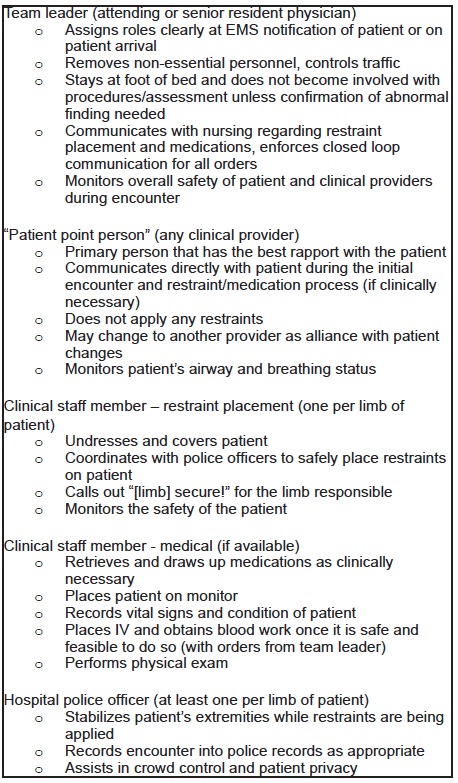
Agitated patient care team: roles and responsibilities. This is set with the model of 2–3 nurses, 2–3 physicians, and 2–3 police officers, and 1–2 ancillary staff members. *EMS*, emergency medical services; *IV*, intravenous

Our educational team derived a 30-minute introductory interactive lecture from core elements of validated aggression management courses. Key components of the didactics including crisis management principles, de-escalation techniques, and proper application of restraints were summarized in a pre-session handout that was distributed to our learners prior to beginning of the session. Moreover, ED leadership constructed formal roles and responsibilities prior to the didactic session. These roles were described in detail in the pre-session handout and re-enforced with staff during the didactic session (see Figure 1 for detailed description). At the end of the didactic component, we solicited participating staff for quality improvement initiatives that could be implemented in the clinical setting to further advance staff and patient safety during treatment of the patient with a behavioral emergency.

### Immersive Simulated Encounters

We recruited healthcare professionals to act as SPs and trained them in conjunction with our content experts to simulate two agitated patient scenarios typically encountered by ED staff members. The course participants were expected to use the de-escalation techniques in an interprofessional manner discussed during the didactic session to calm the simulated agitated patients. The simulations were designed so that de-escalation techniques would only be partially successful, and the team would then need to apply physical restraints and medical therapy to complete the scenarios. A code phrase, “mickey mouse,” was designated as a “time out” should participants or SPs feel that they were in physical danger or out of their comfort zone during the scenarios, while the educator team closely monitored each simulation encounter from the control room. At the completion of each immersive simulation, the interprofessional group of participants immediately proceeded to a structured debriefing session led by health professions educators specifically trained in educational theory and debriefing concepts. We ensured that the main discussion points focused on participant attitudes towards factors contributing to patient aggression as well as interprofessional collaboration and communication skills demonstrated during the encounters.

### Case 1: Intoxicated Patient with Head Trauma

The first scenario involved a patient who was brought to the ED by paramedics for evaluation of altered mental status and minor head trauma. The patient appeared to be intoxicated with alcohol and became angry and threatening during the triage process. The participants were required to use the de-escalation techniques demonstrated during the didactic session, recognize that the patient had head trauma as evidenced by a scalp laceration, order a head computed tomography and diagnose a subdural hematoma that was due to an acute fall from a standing position. Discussions focused on workflow and restraint placement as a large consumption of manpower and resources, especially on weekend overnight shifts when many intoxicated patients presented simultaneously in the ED.

### Case 2: Psychiatrically ill patient with sympathomimetic toxidrome

The second scenario featured a physically and verbally aggressive patient with underlying psychiatric illness who initially responded to de-escalation by staff. However, he quickly became more aggressive and dangerous despite participants’ attempts. He was later found to have ingested phencyclidine, requiring medical therapy and safe restraint placement. Facilitators often needed to intervene during this second simulation to halt the scenario and correct participant errors, using a strategy similar to “rapid cycle deliberate practice” training.^24^ We noticed potential real physical danger to the participants or the SP due to the physical nature of the case. Staff often raised concerns regarding the durability of the restraints and specific mechanical details of the restraint placement process to prevent injuries, which were clarified by our educators and CMT experts.

### Implementation Strategies

Engaging and securing administrative support was key to the successful implementation of our intervention. To minimize disruption of clinical care, sessions were incorporated within already established training time periods for nurses and resident physicians. For nursing, we incorporated this course into their annual competency training. Simulation didactics for the residents were scheduled on a weekly basis in the simulation center and 10 of those sessions were used for this course.

## IMPACT

### Survey Results & Staff Response

**Figure 2:**
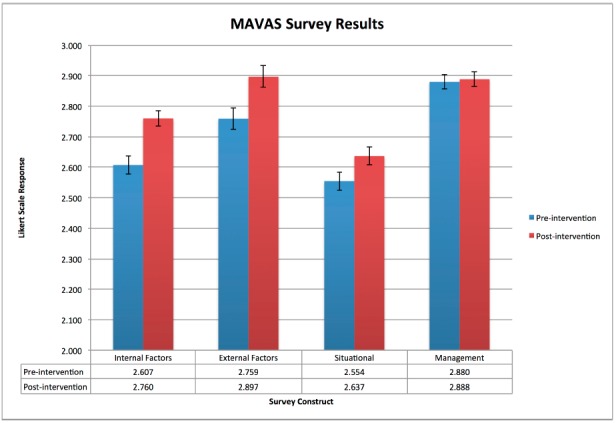
Management of Aggression and Violence Attitude Scale (MAVAS) survey results showing emergency department personnel’s changes in attitude to patient aggression after participation in course on managing agitated patients.

In total, we conducted 10 three-hour sessions from July to September 2014. One hundred sixty-two ED staff members completed the course with >95% staff participation, generating a total of 106 paired pre-post surveys. See the Table for a detailed list of survey respondent demographics. Constructs for internal factors, external factors and situational/interactional perspectives on patient aggression significantly improved post-intervention (p<0.0001, p<0.002, p<0.0001 respectively, Figure 2). Staff attitudes toward management of patient aggression did not significantly change (p=0.542). Secondarily, staff participants gradually generated a list of quality improvement initiatives as the weeks went by, many of which were successfully implemented including the creation of an ED-based interprofessional crisis management alert and response protocol.

The results of the MAVAS survey reflected our staff participants’ immediate changes in attitudes toward patient aggression factors as a result of our course except in the construct of clinical management of aggression. As our curriculum objectives focused heavily on prevention and recognizing factors contributing to aggression rather than the specific medical management of aggression, the survey accurately reflected our intended interventions. In fact, we wished to deliberately not discuss details of clinical decision-making while caring for our targeted population for the purposes of this course. Our agitated ED patients present with a breadth of medical and psychiatric etiologies, and management depends heavily upon the unique circumstances and ultimate diagnoses of a particular patient encounter.

Staff participants overwhelmingly endorsed and welcomed the SPs in the hands-on components of the course and frequently commented on how having SPs in the simulations significantly increased fidelity and helped recreate a realistic scenario for them. Many in fact forgot that they were participating in a simulation altogether and experienced the same fear, anxiety and frustrations that they felt while caring for an agitated patient in a prior clinical shift. Although none of the SPs or learners used the “time out” even once or suffered any injuries during the course, we paid close attention to the play of the scenarios in the control room to observe for latent safety lapses. As mentioned above, educators entered the simulation room on multiple occasions to pause the scenario and intervene in the second case with the acutely aggressive patient.

### Pitfalls and Limitations

This course was time and resource intensive for the instructors. Even with an average of 15–20 learners per session, we required 10 sessions to completely train our department. Each session required at least nine to ten instructors and assistants to run the simulations and lead the interprofessional debriefing sessions. We found that having a core team of nursing, physician and police educators, at least one of whom was trained in immersive simulation and debriefing structure and techniques, was critical to ensuring continuity and consistency between sessions. Our program benefited from the availability of a robust simulation center at our institution. We believe that other educators and administrators interested in implementing this curriculum can still successfully conduct the simulations and debriefing sessions in small meeting rooms or other office spaces within a hospital or learning environment. With regards to program evaluation, the cohort of educators and researchers for our pilot study were also in leadership positions within the department, which may have confounded our participants’ responses to the MAVAS survey.

### Future Directions

Additional work includes longitudinal data collection of staff attitudes over longer time periods, comparison of different methods of training and curriculum design, as well as a higher level of evaluation in the definitions of translational educational research to include patient outcomes or direct indices of care safety and quality.^18^,^25^ Finally, validation studies of our interprofessional curriculum across different clinical sites may expand the applicability of the training methodology used in our study to a wider spectrum of institutions and departments.

In order to promote sustainability, stricter implementation of the defined roles and quality improvement initiatives need to occur on a consistent basis with buy-in from administration and staff members across all professions. A qualitative analysis of ongoing barriers and staff concerns to sustaining these efforts and caring for our agitated patient population may bring more key issues to light. Finally, the curriculum can be re-enforced with repeat sessions at scheduled intervals with shorter didactics or targeted to new staff hires and incoming physician trainees.

## CONCLUSION

An interprofessional simulation-based team-training curriculum successfully improved staff attitudes toward the factors impacting the care of patients with behavioral emergencies in the ED. We hope the next steps in interprofessional education research will lead us toward sustainable and outcomes-based measures to improve patient and staff safety utilizing team effectiveness in caring for the potentially aggressive patient.

## Supplementary Information



## Figures and Tables

**Table:** Survey respondent demographics.

Characteristic	N
Staff clinical role	
Ancillary staff	6
Nurse	43
Physician	36
Hospital police	21
Gender	
Male	44
Female	62
Age group	
21 to 25	2
26 to 30	36
31 to 35	14
36 to 40	13
41 to 45	12
46 to 50	9
51 to 55	11
56 or older	9
