# Supplementary material for: Coordinating a Team Response to Behavioral Emergencies in the Emergency Department: A Simulation-Enhanced Interprofessional Curriculum
Source: West J Emerg Med. 2015 Oct 22;16(6):859–65. doi: 10.5811/westjem.2015.8.26220 (PMC4651583; doi:10.5811/westjem.2015.8.26220)
Supplement: Supplementary file 1 [file wjem-16-859-s001.pdf]

CODE ID:_________________________ CIRCLE: PRE POST

The Management of Aggression and Violence Attitude Scale (MAVAS)

- The purpose of this questionnaire is to obtain your perceptions of aggression and the management of aggression at this facility.
- For each item, please darken the circle that reflects your opinion about the statement. For example:
  ○ ○ ● ○
- If you wish to change your rating, place an X completely through the circle and then darken another circle.
  ● ○ ⊗ ○
- This is an anonymous survey. Do not write your name on this form.
- The questionnaire contains 30 items and takes about 10 minutes to complete.

		Strongly Agree	Agree	Disagree	Strongly Disagree
1	Patients are aggressive because of the environment they are in.	○	○	○	○
2	Other people make patients aggressive or violent.	○	○	○	○
3	Patients commonly become aggressive because staff do not listen to them.	○	○	○	○
4	Gender mix on the wards is important in the management of aggression.	○	○	○	○
5	It is difficult to prevent patients from becoming violent or aggressive.	○	○	○	○
6	Patients from particular cultural groups are more prone to aggression.	○	○	○	○
7	Patients are aggressive because they are ill.	○	○	○	○
8	Poor communication between staff and patients leads to patient aggression.	○	○	○	○
9	There appear to be types of patients who frequently become aggressive towards staff.	○	○	○	○
10	Cultural misunderstandings between patients and staff can lead to aggression.	○	○	○	○
11	Different approaches are used on this ward to manage patient aggression and violence.	○	○	○	○
12	Patients who are aggressive towards staff should try to control their feelings.	○	○	○	○
13	When a patient is violent, seclusion is one of the most effective approaches to use.	○	○	○	○
14	Patients who are violent are often restrained for their own safety.	○	○	○	○
15	The practice of secluding violent patients should be discontinued.	○	○	○	○

CODE ID:________________________ CIRCLE: PRE POST

		Strongly Agree	Agree	Disagree	Strongly Disagree
16	Medication is a valuable approach for treating aggressive and violent behaviour.	○	○	○	○
17	Aggressive patients will calm down automatically if left alone.	○	○	○	○
18	The use of negotiation could be used more effectively when managing aggression and violence.	○	○	○	○
19	Restrictive care environments can contribute towards patient aggression and violence.	○	○	○	○
20	Expressions of aggression do not always require staff intervention.	○	○	○	○
21	Physical restraint is sometimes used more than necessary.	○	○	○	○
22	Alternatives to the use of containment and sedation to manage patient violence could be used more frequently.	○	○	○	○
23	Improved one to one relationships between staff and patients can reduce the incidence of patient aggression and violence.	○	○	○	○
24	Patient aggression could be handled more effectively on this ward.	○	○	○	○
25	Prescribed medication can in some instances lead to patient aggression and violence.	○	○	○	○
26	It is largely situations that contribute towards the expression of aggression by patients.	○	○	○	○
27	Seclusion is sometimes used more than necessary.	○	○	○	○
28	Prescribed medication should be used more frequently to help patients who are aggressive and violent.	○	○	○	○
29	The use of de-escalation is successful in preventing violence.	○	○	○	○
30	If the physical environment were different, patients would be less aggressive.	○	○	○	○

Role:

- Ancillary staff ❑
- Nurse ❑
- Physician ❑
- Hospital Police ❑

Gender:

- Male ❑
- Female ❑

Age Group:

- 20 or under ❑
- 21 to 25 ❑
- 26 to 30 ❑
- 31 to 35 ❑
- 36 to 40 ❑
- 41 to 45 ❑
- 46 to 50 ❑
- 51 to 55 ❑
- 56 or older ❑

Thank you for completing this survey.

CODE ID:________________________ CIRCLE: PRE POST

MAVAS Sub Scales (for internal use only)

**Internal**	**Score (mm or cm)**
**Q4**	--------
**Q5**	--------
**Q7**	--------
**Q9**	--------
**Q14**	--------
**Q17**	--------
**Total**	--------

**External**	**Score**
**Q1**	--------
**Q16**	--------
**Q27**	--------
**Q24**	--------
**Q25**	--------
**Q26**	--------
**Total**	--------

**Situational/Interactional**	**Score**
**Q2**	--------
**Q3**	--------
**Q6**	--------
**Q15**	--------
**Q20**	--------
**Q21**	--------
**Q22**	--------
**Q23**	--------
**Total**	--------

**Management**	**Score**
**Q8**	--------
**Q10**	--------
**Q11**	--------
**Q12**	--------
**Q13**	--------
**Q18**	--------
**Q19**	--------
**Total**	--------
